# Embodied Songs: Insights Into the Nature of Cross-Modal Meaning-Making Within Sign Language Informed, Embodied Interpretations of Vocal Music

**DOI:** 10.3389/fpsyg.2021.624689

**Published:** 2021-10-22

**Authors:** Vicky J. Fisher

**Affiliations:** Multimodal Language and Cognition, Max Planck Institute for Pyscholinguistics and Centre for Language Studies, Radboud University, Nijmegen, Netherlands

**Keywords:** sign language, embodiment, analogy structure mapping, Conceptual Metaphor Theory (CMT), cross-modal correspondences, embodied and grounded cognition, dance and expressive movement, music

## Abstract

Embodied song practices involve the transformation of songs from the acoustic modality into an embodied-visual form, to increase meaningful access for d/Deaf audiences. This goes beyond the translation of lyrics, by combining poetic sign language with other bodily movements to embody the para-linguistic expressive and musical features that enhance the message of a song. To date, the limited research into this phenomenon has focussed on linguistic features and interactions with rhythm. The relationship between bodily actions and music has not been probed beyond an assumed implication of conformance. However, as the primary objective is to communicate equivalent meanings, the ways that the acoustic and embodied-visual signals relate to each other should reveal something about underlying conceptual agreement. This paper draws together a range of pertinent theories from within a grounded cognition framework including semiotics, analogy mapping and cross-modal correspondences. These theories are applied to embodiment strategies used by prominent d/Deaf and hearing Dutch practitioners, to unpack the relationship between acoustic songs, their embodied representations, and their broader conceptual and affective meanings. This leads to the proposition that meaning primarily arises through shared patterns of internal relations across a range of amodal and cross-modal features with an emphasis on dynamic qualities. These analogous patterns can inform metaphorical interpretations and trigger shared emotional responses. This exploratory survey offers insights into the nature of cross-modal and embodied meaning-making, as a jumping-off point for further research.

## Introduction

Music, dance and other non-linguistic human movement can involve rhythm, flow, intensity and other expressive qualities, as well as employing internal patterns and structures. In these ways, movement can be innately “musical” without sound, and can both invoke imagery and evoke emotional responses.^1^ These shared properties come to the fore when human movement is used to make sound visible, which is at the heart of embodied song interpretation. The visual musicality of movement can be harnessed to transform modally irrelevant representations of features into an accessible form for d/Deaf audiences.^2^

A song is a gestalt—an integrated entity (object, structure or experience) in which the whole is experienced as something greater than, or different from, the sum of its parts. Meaning and affect are established through the specific combination of patterned words, rhythm, melody, harmonies, and instrumentation, supplemented by a range of non-acoustic features such as gestural body movements and dance, performers’ personalities, and visual elements including clothing and lighting.^3^ To transform a song into an embodied-visual rendition for d/Deaf audiences, an interpreter has to decide which elements to include, aiming to capture and re-present the features that are most salient in constructing an alternative gestalt that effectively communicates the “message,” without overwhelming viewers with too much information. To do this, they need to employ what they deem to be the most effective strategies, across modalities.

Well over a hundred videos of sign language informed, embodied interpretations of vocal music are available on YouTube, and Dutch sign language interpretations were broadcast in the Netherlands as part of the 2019 and 2021 Eurovision Song Contests. There are training programmes available in various countries, including the Netherlands and United States (see Table 1: list of web resources). A wide range of practices exist, many of which share a number of core characteristics, but also contain substantial diversity and individuality.^4^ Variation exists in terms of hearing status and sign language expertise of both the practitioners and target audiences; guiding principles and intentions, such as communication, access and/or art; and the embodiment techniques that are employed. The common factor is that all these practices combine features of a sign language^5^ with other bodily movements and gestures in order to create an embodied, visual representation of vocal music.^6^ This paper will use the umbrella term **embodied song** interpretations, except where specific practices are under discussion.

**TABLE 1:** List of web resources including cited videos.

Cited videos (publicly available)			
Artist/Contributor	Title/Work	URL	Notes
Galloway Gallego, Amber	This is Me (Greatest Showman)	www.youtube.com/watch?v=jo4LL8M31eI	ASL
Stolk, Mirjam	This is Me (Greatest Showman)	www.youtube.com/watch?v=hyygU0j_t3w	NGT
Stolk, Mirjam	Psycho (Muse)	www.facebook.com/100193003430942/videos/628924 037810430	NGT
Tubert, Sarah	Show Yourself (Frozen)	www.youtube.com/watch?v=5ywnOWWfOWM	ASL
Tubert, Sarah	This is Me (Greatest Showman)	www.youtube.com/watch?v=fFMCFXWbJrA	ASL
Uittenbogert, Tom	Together (Ryan O’Shaughnessy)	www.youtube.com/watch?v=GgFaA0bDD SI&feature=youtu.be	NGT

**YouTube channels** (indicative list)			

**Name**		**URL**	**Notes**

Amber Galloway Gallego		www.youtube.com/user/1stopforasl	ASL videos and vlogs
Sarah Tubert		www.youtube.com/user/et15phonehome	ASL videos and vlogs
Mirjam Stolk		www.youtube.com/user/MirjamSTolkNGT	NGT muziektolk videos
Hanneke en Mirjam [de Raaf and Stolk]		www.youtube.com/channel/UC9WliDzFCX3ow EtoGutZoMg/featured	NGT muziektolk videos

**Websites** (indicative list)			

**Site name/URL**		**Notes**	

www.academievisuelemuziek.nl/		Visual Music Academy—information about courses in visual music and sign dance (in Dutch; Director: Tom Uittenbogert)	
www.aslmusiccamp.com/		Information about ASL song signing training	
www.handspeak.com/		An online, searchable dictionary of ASL	
www.muziektolken.nl		Information about + register of qualified Dutch song signers (in Dutch; Directors: Mirjam Stolk and Hanneke de Raaf)	

This paper acknowledges but does not focus on the social or political discussions around Deaf culture and the important issue of cultural appropriation with regards to sign language use in music visualizations, by and for d/Deaf people (for discussion of this issue see, for example: Lisi, 2014; Torrence, 2014). It recognizes that the involvement of d/Deaf practitioners and researchers is essential in ensuring that d/Deaf insights and perspectives are foregrounded.

Songs that combine vocals and instrumental music exist within an acoustic-temporal domain and are perceived primarily aurally (sometimes supplemented by spatio-temporal information such as during live performances or music videos)—where a distinction is required, songs in this modality will be referred to as acoustic songs. Embodied songs are spatio-temporal and are perceived visually, however, embodied songs are predominantly presented alongside a live music performance, music video or are accompanied by the sound-track. The primary goal is to increase access for d/Deaf audiences to the non-visible expressive elements of a song and the associated cultural experience. As a by-product, additional layers of information in the embodied-visual modality emerge for hearing viewers.

There is currently no explicitly formulated hypothesis regarding the nature of embodied song interpretations of vocal music and their relationship to meaning making. Music scholar Anabel Maler has proposed that in American Sign Language (ASL)-based song signing, linguistic signs are integrated with “non-music-producing gestures that can take the place of sound” (2013, p. 5.2). She further argues that “For the deaf viewer, the knowledge that there is music playing and that [the performer’s] movements seem deliberate and dance-like is sufficient for understanding that there is a degree of conformance between the music and the signer’s gestures.” (2013, p. 3.7). In this paper I focus on a range of strategies that interpreters employ in embodied song production, drawing on a range of theories within a grounded cognition framework to consider in what ways the transformations may go beyond an implication of conformance to communicate deeper, more meaningful information for d/Deaf audiences. I consider what analogy mapping and cross-modal correspondences theories may reveal about the nature of meaning within embodied song practices through unpacking the relationship between particular movements, sounds, images or ideas, and emotions. In this way I begin to unpack how interpreters communicate the underlying message of the song to their audience, which is a central goal according to the contributing experts.

The notion of an “underlying message” is recognized as being rather vague and it is not claimed that a song has a single objectively measurable message. Relevance theory (Sperber and Wilson, 1986) and the concept of motivated meaning making (Taub, 2001, p. 9) are particularly helpful here in understanding that each individual will understand and in fact perceive a source input based on their own prior knowledge and experiences, and the framing that they bring to the encounter. “Underlying message” implies a degree of communicative intention and is linked to the broader complex concept of “meaning,” taken here to encompass diverse aspects of conceptual understanding and emotional responses.

Four inter-linked types of embodied song meaning are considered—the meaning of the embodied-visual signals in relation to^7^ :

1. musical form;
2. sensory-motor, concrete referents or concepts;
3. abstract concepts;
4. emotional responses.

The core or central message of a song can often be summarized in a single sentence, such as “This is Me” (Pasek and Paul, 2017) is about overcoming negative forces to claim ownership of one’s own identity” (author’s interpretation). However, the full effect is achieved through the combination of specific words and music. In transforming the whole package into an embodied, visual rendition, an interpreter selects the features that they identify as being most significant and re-presents them using the tools and strategies at their disposal. This paper looks at what those strategies are and how these may help establish meaning for the viewer.

To achieve this, I held discussions with practitioners of embodied song interpretation in the Netherlands and analysed various publicly available recordings of interpretations. I have drawn together a diverse range of theories and practices in associated fields of study, to inform an exploration of the relationship between acoustic and embodied-visual versions of songs and what this may tell us about meaning-making, as a jumping off point for targeted studies. Research areas (citations for which appear in section “Background: Grounded Cognition, Semiotics, Conceptual Metaphor Theory, Analogy Mapping, and Cross-Modal Correspondences”) include embodied and grounded cognition, cognitive linguistics (especially Conceptual Metaphor Theory), cross-modal correspondences, sign language research, sign song, sign poetry, co-speech gesture, music embodiment, dance, and inclusive practices. It is hoped that the paper will be of value for each of these and in particular for people who are interested in the intersections between them.

Section “Background: Grounded Cognition, Semiotics, Conceptual Metaphor Theory, Analogy Mapping, and Cross-Modal Correspondences” presents background theory that can help unpack the transference of information across modalities. Section “Embodiment Strategies—Descriptive Survey and Theoretical Discussion” applies these theories to the embodiment strategies used within song interpretation. Section “Interpreters” Identities as Broad Context” considers the issue of individual differences between interpreters and how this may impact on embodied song production. The paper concludes with a general discussion and suggestions for future studies, in section “General Discussion.”

## Background: Grounded Cognition, Semiotics, Conceptual Metaphor Theory, Analogy Mapping and Cross-Modal Correspondences^8^

In embodied songs, a great deal of meaningful information is communicated through sign language presentation of the lyrics enhanced by expressive modifications. This is augmented by embodiment of the accompanying music. The focus of this paper is on the para-linguistic expressive modifications of core semantic units, and the embodiment of musical features.^9^

I will begin by outlining some principles that may underpin the different ways in which a unit of information in one modality (e.g., acoustic feature) can be meaningfully represented in another modality (e.g., embodied-visual feature). Some of these also influence modifications of the linguistically encoded elements. I will explore the notion of **mapping**, a mechanism by which two or more entities can be understood as being equivalent. This will be considered in relation to **semiotics**, **analogies** and **Conceptual Metaphor Theory** (CMT). I will also present the related concept of **Cross-Modal Correspondences** (CMC), the connections between signals in different modalities that are either associated with the same information source (e.g., “big objects” and “loud noises”) and/or are understood as being in some way equivalent to each other (e.g., “big” and “loud” more generally). Each of the theories have something to offer our understanding of embodied song interpretations and will be used to unpack the strategies that are discussed in section “Embodiment Strategies—Descriptive Survey and Theoretical Discussion.”

Semiotics, CMT, analogy mapping and CMC are rooted in the grounded/embodied cognition paradigm (Barsalou, 2008; Bläsing, 2020), which holds that human understanding is founded upon cognitive processing of experiences that are perceived through sensory-motor input—we learn about ourselves and the world around us through our internal sensations of movement and our interactions with our environment.^10^ We see, hear, taste, smell and both touch and physically manipulate our surroundings—or the combination of these inputs that are available to each of us. We also have affective and emotional responses to sensory-motor inputs. Making sense of new experiences involves analogizing, that is, comparing new experiences to prior ones, establishing in what ways they are similar and transferring information from one to the other.

### Semiotics

Signals in different sensory modalities presented with communicative intent, such as a phrases in speech and sign language (**signifiers**, in Saussurian semiotic terminology) are representations of an idea or concept (the **signified**) (Stam et al., 1992, p. 8). An entity in the world, to which both the signifier and signified relate, is known as a **referent**. According to Conceptual Metaphor Theory (CMT) [first proposed by Lakoff and Johnson (1980)], a concept can be represented through any modality. Concepts may refer to concrete entities (such as “a chair”) or abstract ideas (e.g., “something that supports weight” or “support”) and many concepts are shared between diverse referents. For example, the concept of a “wave,” which at its most abstract level is a regular, repeating increase and decrease of energy, can be manifest in fluctuating musical pitch or volume, and the up and down motion of an arm (each of which can be both signifiers and referents). Each individual’s understanding of “wave” is informed by all their experiences of different forms of waves (potential referents) which allows for the transfer of information between one form and another. Thus, a fluctuating pitch is like an undulating arm is like the sea lapping at the shore or a storm in the ocean—contingent on the particularities of features such as instrumentation, spatial size and dynamic qualities.

Peircian semiotics identifies three types of sign, defined by their relationship to their referents: **icon, index** and **symbol** (Aston and Savona, 1991, p. 6). An icon shares similarities of form with the referent, such as a photograph “looking like” the object to which it refers, sharing visual features. An index “points to” a referent through some innate or established, possibly causal connection, such as smoke indicating fire. Symbols are signs in which the signifier refers to a referent (and the signified concept) through convention.

In general, spoken languages use primarily symbolic signs with some iconic characteristics such as those revealed by sound symbolism (see below). Linguistic syntax (grammatical structure) can also have an iconic relation to meaning, such as the ordering of events in a discourse reflecting the temporal order of their referents. Sign languages largely have a more iconic relationship to their referents predominantly because the majority of entities that we experience in the world have visuo-spatial and kinaesthetic characteristics that we can represent with our bodies (Taub, 2001, p. 61).

Embodied-visual iconic signs provide additional information to graphic-visual ones, because of the felt sensations—the kinaesthetic experience of “up” is not simply spatial—to achieve “up-ness,” involves a journey, a way of moving and provides us with different experiences of ourselves and the world, to the experience of “down-ness.”^11^

Musical relations to referents are primarily iconic, potentially sharing similarities of sound but often reflecting underlying structural characteristics as will be discussed below.^12^ Dance and other non-linguistic movement can employ both intra-modal (e.g., visual-to-visual) and more form-oriented, cross-modal iconicity. Embodied songs use embodied-visual signifiers to re-present information parsed from acoustic signals and may bear an iconic resemblance to their source and to other analogical referents. The comparison of features of one entity to those of another is known as **mapping** which is considered in the following section.

### Iconicity Types, Metaphor and Analogy Structure Mapping

In the context of gesture and sign language research, iconicity refers to mapping in which representations “mimic the sensorimotor attributes of a referent” (Ortega and Özyürek, 2019: 1) such as spatial and qualitative features (Müller, 1998). In embodied representations, iconicity usually employs one of four “modes of representation”: Tracing, holding/touching, enacting, and embodying.^13^ The latter two are the most common within embodied song practices and are the most adept at communicating musical qualities:

◼ ENACTING—performing a functional movement such as playing a piano or breaking something with one’s hands. In performative contexts such as dance, some features of enacted everyday movements are altered or exaggerated, such as making the movement larger or dynamically sharper (Fisher, in press).^14^
◼ EMBODYING–the moving body “stands in” for the entity that it represents such as water, or the melodic line of a song.^15^

In gesture and sign language, iconicity is most commonly associated with visuo-spatial features of a referent, such as its shape or movement in space. Less attention has been paid to dynamic, effort or qualitative features. Beyond the movements of instruments and performers, music does not have visible spatial form, therefore, in embodied songs, iconicity is most likely to involve temporal and qualitative characteristics. That is, both the acoustic signals and the embodied-visual ones into which they are transformed, will communicate a shared underlying energy pattern.

Effort qualities (also known as “dynamics” or “dynamic qualities”)^16^ refer to the way in which movement is performed and includes things like muscle tension. There are systems for analysing and talking about dynamics, such as Laban Movement Analysis (LMA), which may be useful for unpacking the nature of embodiment. LMA identifies four aspects of effort, each with two elements: Weight (strong-light), Time (sudden-sustained), Space (bound-free) and Flow (direct-flexible) (Laban and Ullmann, 2011). This provides a basic framework for analysing this visceral feature of bodily action.

Mapping can also take place between non-sensory-motor and abstract concepts. Abstract concepts are often general categories that emerge from prior direct experiences, such as PROGRESS being abstracted from numerous actions that take us from one position or “state” to another.^17^ Mapping often occurs between a concrete exemplar and the abstract idea, in which the relations between the physical features of the concrete SOURCE, perceived through our sensory-motor systems, are carried over to the more abstract TARGET. This is the basis of metaphorical transfer in Conceptual Metaphor Theory, which refers to the underlying mapping between conceptual domains, and is often framed with the construction TARGET IS SOURCE (Taub, 2001, p. 94–95; Lakoff and Johnson, 1980). For example, PROGRESS IS (INCREMENTAL) DIRECTIONAL CHANGE—which can be expressed, for example, by symbolic, linguistic labels (spoken or signed), embodied movement toward a goal, or in the resolution of a melodic line. The relationship between the latter two expressions are iconic in the semiotic sense that they share similarities of (structural) form but is referred to as metaphorical in gesture and sign language literature because it does not involve sensory-motor similarities (Cooperrider and Goldin-Meadow, 2017).

Sensory-motor mapping may occur at the level of attributes, that is, the basic characteristics of entities such as shape, color or acoustic tone. However, mapping can also occur at a structural level (regardless of entities’ sensory-motor or abstract nature), which involves the ways that attributes relate to each other and the role that they play within the whole. In the PROGRESS examples offered above, the attributes of the entities of a human body and musical notes are dissimilar but both undergo directional change, “traveling” from a starting “position” toward a “goal.” It is the ways that the start and end states relate to each other within each system, that is comparable. Mapping that foregrounds relational structures supports the transfer of knowledge from one entity to another and this is what characterizes an **analogy**, according to Gentner’s Structure Mapping framework (1983; 1988, p. 48). When analogies occur across different modalities, the attributes are largely dissimilar and comparisons between internal relationships become key.

Sarah Taub has proposed a structure mapping based Analog-Building Model that unpacks how iconic and metaphorical signs are constructed in sign languages (Taub, 2001, p. 43–55). Encoding of abstract, metaphorical concepts that do not have an innate visuo-spatial form, employs double-layered mapping via a concrete concept that exemplifies the metaphor, to an iconically encoded sign with a physical form that retains important roles and relations (Taub, 2001, p. 97). For example, in ASL, GROW can be represented by a TREE form emerging from the GROUND. An encoded form can be modified and manipulated according to the constraints of the language, such as in pluralization. Variations to established schematized forms generate specific meanings. For example, different types of both concrete and metaphorical GROWTH emerge from “shooting up vertically” vs. “following a meandering, twisting path.” Thus, specific aesthetic, communicative contexts may inspire modifications of form that go beyond established linguistic rules. In the context of songs, such subtleties of meaning may not be explicit in lyrics but are suggested by the poetic, expressive and musical framing.

Metaphors are not always uni-directional, tied to a concrete SOURCE/abstract TARGET, or limited to only two entities at a time (see Brandt, 2009, p. 46–49 and Brandt and Brandt, 2005) for a discussion of Conceptual Integration Theory, Conceptual Blends and Relevance Theory). Multi-directional, multi-layered analogs and metaphors abound in music and dance, which are primarily abstract artforms in which meaning arises from internal relational structures and ways in which those structures are perceived as being analogous to entities experienced in the world.

Emotions are often in the target position in conceptual metaphors in that they are conceptualized through associated physical experiences. Emotions are generally considered to be abstract concepts because they cannot be directly experienced through our externally interfacing senses and do not have a “physical” form. Rather, they are internally felt sensations and can be triggered by, or associated with, sensory-motor experiences such as feeling physically heavy and collapsed inward when we are sad—SAD IS DOWN/HEAVY. Affect (the experience of emotion) can be expressed in different modalities for example, the feeling of sadness may be encapsulated by slow, legato (smooth) music in a minor key or slow, weighty movements (Leman, 2008, p. 21–22). This shared resonance of both music and dance/bodily movement evoking the same emotions, allows one representational form to stand in for and be mapped to the other. However, the emotion itself is not structurally part of the mapping but can rather be triggered by analogous experiences.

Structure mapping provides a valuable framework for unpacking the meaningful relationship between entities. This can integrate with the notion of **cross-modal correspondences**, which specifically considers how signals in one modality may be associated with signals in another.

### Cross-Modal Correspondences

CMC predominantly refer to common pairings between information in different modalities that are associated with the same *object* or *event* (hereafter used interchangeably with “*entity”*). Interrogating the origin of the relationship between signals in different modalities can provide insight into how conceptualization across modalities can be grounded in sensory-motor experience. Well studied pairings include “sound symbolism”—the associations between sound and size/shape (such as the bouba/kiki effect (Ramachandran and Hubbard, 2001)), loudness-brightness and pitch-brightness/spatial elevation/sharpness (see Spence, 2011, p. 974–75).^18^ There are three main overlapping forms of correspondences: **statistical, structural** and **semantically mediated** (Spence, 2011, p. 978), which may all be relevant to embodied song practices.

**Statistical correspondences** are those that emerge from associations that occur in the world—different sensory-modal information arises from a single event or entity. For example, particular resonant frequencies (aural) of an object such as a drum, emerge because of its physical form, its size/mass and shape (visual/tactile). The relation between the modal information becomes established in our memories through repeated exposure.

Spence notes a distinction between *amodal* and *modal* features of an entity. Amodal features are those that can be marked by signals in different sensory modalities, examples of these include duration, rhythm and intensity. Modal features or attributes are those which are intrinsically tied to a sensory modality, such as tonal pitch (Spence, 2011, p. 973). This suggests that correspondences may arise from joint association with either shared (as in marking divisions of time) or “distinct but connected” (such as size of a resonant chamber and the tone of sound it produces) features of an entity.

Statistical correspondences can thus take a number of forms:

**Co-occurrence**: attributes that coincide temporally or spatially but do not have an informative relationship with each other, for example the *color* and *texture* of wood
**Causal relationship:** the form of one feature (that can be experienced through particular modalities e.g., *size/shape*) gives rise to another feature (such as *tone/timbre*) as with a drum.
**Shared amodal referent:** different ways of signaling or marking the same feature in either integrated or distinct events (e.g., visual and acoustic marking of patterned divisions of time i.e., *rhythm*).

**Structural correspondences** are those arising from the way our perceptual systems are organized, which enable us to perceive patterns and contrasts regardless of the input modality (Spence, 2011, p. 978; see also Stevens, 1957).^19^ One concept in which correspondences may arise at a neural level, is “magnitude”—the amount of a particular attribute, such as sound (volume) or size (Walsh, 2003).

**Semantic mediation** Some correspondences may arise from, or at least be reinforced by, shared terminology, rather than exposure to phenomena or innate neural associations. The use of the terms “high/low” in reference to both tonal pitch and spatial elevation may be an example of this. If the association is purely arbitrary then substituting one modality for another will not carry with it any intrinsically meaningful information. This is particularly pertinent in the context of embodied songs in which CMC are employed with the intention of communicating meaning rather than indicating a superficial connection. It may, however be the case that semantic mediation primarily serves to strengthen underlying statistical or structural correspondences making some associations more dominant within a culture.

The proposed innate tendency to recognize patterns (i.e., structural correspondences) perhaps enables us to perceive and store examples of these patterns (i.e., statistical correspondences) that we can then call on to communicate ideas to others. In this way, structural correspondences facilitate learning of statistical correspondences, which can be supported and mediated by semantic units.

### Background Theory Conclusion

The theories outlined above, can offer insight into relationships between music, visible movement, concrete referents, metaphorical meanings and emotional responses. It is important to be aware that whilst these theories focus attention primarily on the relationships between entities or experiences, (and the signals that are used to signify them), the **context** in which they exist can be highly influential. Context falls into two main categories, local and broad. The **local context** is information that is spatially and/or temporally present. For an embodied song interpreter, this includes all features of the song and the circumstances in which it will be presented, such as at a concert or on YouTube. **Broad context** is highly variable. It includes personal characteristics and background such as hearing range, musical and dance training, sign language knowledge, personality and much more. Beyond that, there are socio-cultural factors that inform personal context and can have major impact on the representations that are created. Examples of these factors are knowledge of music genres, films and art-works with similar themes, politics, and both linguistic and social cultures.

Section “Embodiment Strategies—Descriptive Survey and Theoretical Discussion” will draw on the preceding theories about how different modalities correspond with each other and how the structural underpinning of these relationships can ensure meaningful communication for d/Deaf audiences, in a uni-modal embodied-visual form.

## Embodiment Strategies—Descriptive Survey and Theoretical Discussion

The strategies presented below are based on the approaches taken by leading Dutch practitioners and trainers, Tom Uittenbogert, André Uittenbogert and Mirjam Stolk (with additional input from Hanneke de Raaff as well as student and bimodal bilingualism researcher, Francie Manhardt), as outlined in private correspondence (2020). The strategies are illustrated with examples from publicly available videos (listed in references and Table 1) and discussed in relation to the theories presented above. The focus will be on features in acoustic and embodied-visual modalities which supplement the meanings suggested by the codified, symbolic signs (words and manual signs). Such features are more integrated in embodied songs than their acoustic counterparts because they are performed simultaneously, by one individual, and draw on the established, expressive lexicon of sign languages. For more detailed discussion of sign language specific elements in song-signing (see Maler, 2013, 2015) as well as overlapping work on sign poetry by Mirzoeff (1995), Blondel and Miller (2001), Russo et al. (2001), and Sutton-Spence (2005).

An effective embodied song interpretation ostensibly uses the original song as a multi-featured score, providing instructions for the interpreter to embody, creating meaning in a medium that is richer and more meaningful to d/Deaf audiences. As will be seen, the demands on an interpreter are highly complex, with a single individual communicating diverse information from different sources, at the same time—a little like a visual “one-man-band.”

The signals from an acoustic song will be considered in two main groups, the lyrics and the musical features. Each sub-section will begin with brief explanation of the song feature followed by a descriptive summary of embodiment strategies used by practitioners, with illustrative examples. This is then followed by a discussion of the types of CMC and analogy mapping that might be involved and how these may ensure the transfer of meaning beyond the lyrics. All analyses are made by the author and are intended to be indicative, providing a starting point for discussion and proposals for further research.

### Lyrics

A direct translation of a song’s lyrics into sign language, as if they were a spoken text, is rarely appropriate for many reasons, both linguistic and aesthetic, including rhythmic patterning, differences in metaphoric framing and the role of iconicity. To create a rich interpretation, practitioners study the lyrics in depth (when possible), aiming to establish a complex understanding of what they perceive to be the underlying message and how it is achieved, including poetic techniques and (metaphoric) imagery. To achieve this, established features of sign languages, including manual signs, facial and bodily expressivity, mouthings and perspective- or role-taking, are modified and enhanced.

#### Manual Signs

Anabel Maler, in her highly informative paper “Songs for Hands” (2013), describes the main ways in which conventional sign language signs are enhanced for poetic and expressive purposes within embodied song interpretations. These grow from the core parameters: (an established range of) handshapes; location and orientation in relation to the “sign-space”; and movement (which includes pathway and dynamic features). These neologisms, also known as “productive signs,” can take the form of constructions of new signs or modifications of existing signs. New signs predominantly rely on iconicity. Alterations to established linguistic signs are often employed to enhance poetic features such as visual rhymes and alliteration. Common examples include a conventional pathway being altered so that it echoes a previous action, or repetition of a particular handshape (Blondel and Miller, 2001, p. 27).

The greater the interpreter’s fluency, the more eloquent and sophisticated their use of sign language will be. The Dutch Visuele Muziek Academie training program includes extensive exploration of poetic manipulations of sign language, whilst paying attention to maintaining clarity of communication.

**FIGURE 1:**
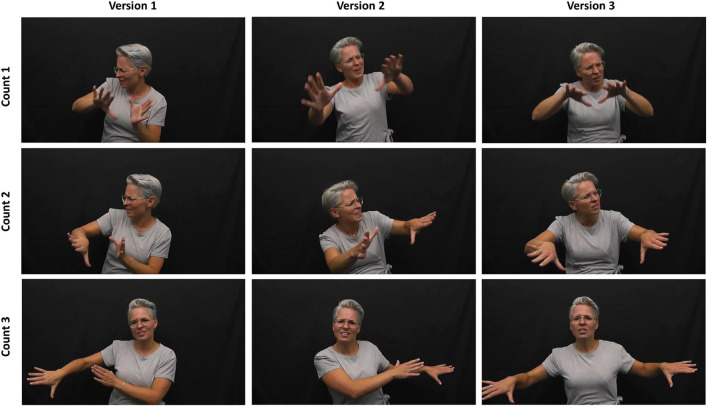
Embodied gestalt construction of meaning, and use of extended time and space—“[drown them] out” versions 1, 2, and 3—at 00:56, 02:11 and 03:10, respectively (Stolk: This is Me—Greatest Showman, 2020).

Action, space and dynamics features (to borrow terminology from dance theory (Adshead, 1988; Laban and Ullmann, 2011; Leigh, 2015) are often layered simultaneously in sign languages, due to the relatively direct, iconic relationship to referents. An example of this is Mirjam Stolk’s embodiment of “I’m gonna send a flood, gonna drown them out” (my emphasis) from “This is Me” (2020) shown in Figure 1. This culminates in a movement in which both hands move as if pushing water away from her body (bound, direct, strong and sustained, in Laban terms), with her final eye-gaze directed at the camera. With this motion, the audience too can “feel”^20^ the pressure (of water) washing something away. The metaphorical framing of the entire phrase is embodied in this single, complex action. The movement shares spatial and qualitative features with its explicit referent (a flood washing away everything in its path) and simultaneously draws attention to its more metaphorical meaning, of the intention to overcome the negative behavior of others (potentially informed by association with the biblical flood—an example of broad contextual knowledge).

Rhythmic patterning of lyrics is discussed alongside instrumental rhythms, below.

**Discussion:** lexical and grammatical forms are mapped between the spoken and embodied languages, with words and signs playing comparable roles in directing attention to shared referents. Embodied representations often have a degree of iconic resemblance to referents with particular attention to effort/dynamic qualities. Poetic features involve cross-modal analogizing, such as rhyming morphemes being mapped to “rhyming” handshapes or spatial pathways. In terms of CMC and analogy mapping the idea of discourse consistent patterning that provides aesthetic cohesion (e.g., rhymes), is represented in distinct, modality-specific ways.

#### Facial and Bodily Expressivity

Lyrics are often performed in a highly expressive, emotionally evocative manner, involving both the body and voice. In sign languages, emotional expression can be directly demonstrated through the body and, as in the example above, this may be exaggerated in artistic and performative contexts through the effort qualities used by hands, whole body and face (Blondel and Miller, 2001, p. 26).

Expressive use of the face is very important in many sign language systems (Pfau and Quer, 2010) and is often used (consciously or otherwise) to add emotional markers such as suggesting distaste or joy, that may not be evident in the manual signs in isolation. Pfau and Quer point out that it is important to differentiate between aspects of facial expressions that play a linguistic role and those that are predominantly affective.

Dynamic qualities such as flow, power and tension, associated with entities in the world and in memories of them, that are invoked by the lyrics, inform modifications to signing in embodied song interpretations. Qualitative features permeate different classes of concepts and can be iconically embodied. They are intrinsic to: actions (e.g., push); physical entities (e.g., water); emotions (e.g., desire), and more abstract ideas such as identity (e.g., “this is me”). As is evident throughout the examples in this paper, dynamic qualities are often brought to the fore in embodied songs.

#### Mouthings

This refers to the lip-sync-like “mouthings” of words, which is an essential part of many sign languages. A range of different strategies may be employed. Some performers mouth all or most of the lyrics in the original spoken language. This can be accompanied by supporting signs, that coincide with the spoken words or short phrases,^21^ or performed simultaneously with fully grammatical sign language phrases that can be understood independently from the vocals. In the latter case sung and signed units do not necessarily coincide temporally (for example Tubert—“This is Me” The Greatest Showman—ASL, 2018). Another alternative is that the practitioner mouths only selected words or phrases, sometimes lip-syncing with the song but sometimes reflecting the grammatical structuring of the signed translation (see Galloway Gallego—This Is Me an ASL interpretation, 2018). The verbal mouthings may not be in the same language as the song, for example, if an English language song is being performed in NGT,^22^ the mouthed words may be Dutch (for example Tom Uittenbogert—Ryan OShaughnessy Together Ireland Gebarentaal ESC2018, 2019).

In instances when an interpreter elects to only mouth key phrases of the lyrics, synchronously with the song, this serves to emphasize the moment and establish it as a core statement. This is evident in diverse versions of “This is Me” in which this titular phrase is consistently mouthed by performers in time with the vocal line, even when other phrases are not. Although mouthings are part of everyday use of sign languages, the song/performance specific choices and modifications indicted above, can impact on meaning-making.

**Discussion:** use of mouthings does not involve analogy beyond “mere appearance” (Gentner and Markman, 1997) but can provide informative local context, such as highlighting or emphasizing key lyrics. In terms of CMC, mouth shape shares a common co-referent with the sound produced (not directly causal in that they must occur in conjunction with actions in other parts of the vocalization system to produce sounds)—both the sound and mouthings are statistically associated with the same words, which symbolically directs attention to a particular concept.

#### Perspective-Taking or Role-Shifts

**FIGURE 2:**
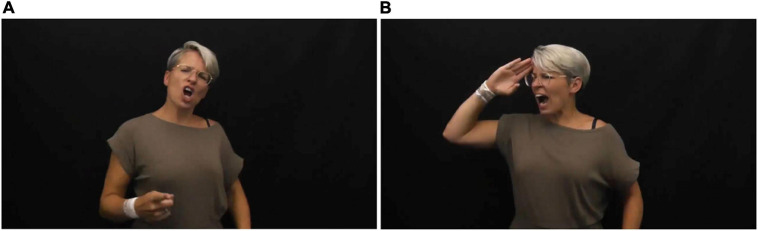
Embodied role-shift/perspective-taking—**(A)** Drill sergeant facing toward the audience and **(B)** Recruit facing downstage-right, in Muse’s Pyscho (Stolk: Pyscho—Muse, 2020).

Another common strategy in sign languages is to differentiate between various “voices” and view-points within lyrics. This is achieved by exploiting different body postures and using the sign space to enact each referent. It is an efficient technique for expressing complex scenarios involving multiple character perspectives (Slonimska et al., 2020). The performative context of song interpretation may encourage the practitioners to fully immerse themselves in characterization beyond that of everyday signing. An example of this is used in Stolk’s rendition of “Psycho” by Muse (2020), in which a shouted dialog occurs between two characters—an army officer and a recruit. Taking the role of the Drill Sergeant, Stolk looks directly at the camera, and asks (signs) “Are you a human drone?”; “Are you a killing machine?”; “I am in control. Do you understand?”—in response to each question, she shifts her body angle and eye-gaze toward down-stage right (diagonally toward the front left edge of our screen) and signs “Aye sir.” The two characters also differ slightly in posture, head angle and facial expression (Figure 2).

**Discussion:** perspective-taking embodies a conceptual feature of the lyrics, making it more visible, that is, it presents a direct embodiment of the idea of distinct roles from a first-person perspective, predominantly using enactment. The interpreter behaves “as if” they are a character in the song narrative—their persona is analogous to the character they portray (Humphries et al., 2021, p. 3)—perhaps in a more exaggerated, performative fashion than in everyday interactions. Cross-modally, different voices (both figurative and literal), are made visible through body-orientation and physical characterization. Interactions between instruments such as “call and response” may also be embodied. Such linguistic-like techniques are a subject of the emerging field of Super Linguistics which applies linguistic theories and methodologies to phenomena beyond language, including gesture, dance and music (Schenkler, 2019; Patel-Grosz et al., 2020, in press).^23^

#### Lyrics Conclusion

There is extensive diversity and complexity involved in transforming lyrics into the visual modality that is built around codified linguistic forms. In attempting to communicate the underlying meaning of the song, decisions are made, particularly in the selection and manipulation of manual signs, which are informed by mapping between the target idea and an embodied representation of it. Many of the strategies discussed here are established elements of sign languages and especially, sign poetry. Expressive features, including use of the face and exaggerated effort qualities are perhaps taken further within this distinctive performative context. Modifications to signed lyrics associated with temporal features, such as rhythmic patterning and structural phrasing, also serve to distinguish embodied songs from other sign language contexts. These will be discussed further in relation to musical features.

### Musical Features

Lyrics utilize predominantly symbolic signs involving semantic units (words and phrases) that have conventionalized referential meanings, both concrete and metaphorical. These meanings are enhanced by musical features. Both music and non-linguistic bodily movement are rarely explicitly referential and meaning emerges through iconic resemblance, structural similarity and the triggering of emotional responses. The embodied-visual signals that are the core communicative units for d/Deaf audiences, potentially have a multi-directional relationship with the acoustic, musical signals, the concepts that they signify and analogous referents that may be evoked, in terms of establishing meaning.

The key musical features noted by the Dutch practitioners that are transformed into bodily movement in embodied song interpretations are rhythm, “intensity,” timbre and instrumentation, and pitch/melody. As will be seen, these rarely exist in isolation but distinct strategies can be identified, which are combined within an embodied song. In general, embodied song interpreters take a top-down approach, starting with their understanding of the concept or message, and using that to intuitively, guide the techniques employed for communication (Manhardt, personal correspondence). Selection of specific components by experienced sign language interpreters, such as particular handshapes, are often highly informed by linguistic constraints, which are part of the context of production.

#### Rhythm and Other Temporal Features

**FIGURE 3:**
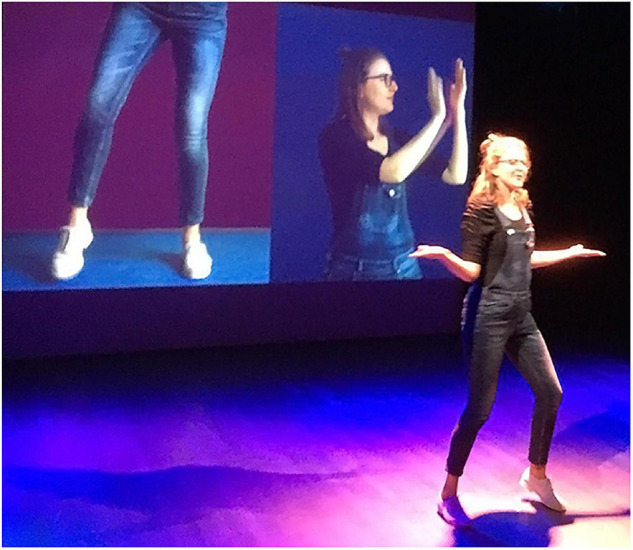
Embodied rhythms—pervasive musical rhythms are indicated through foot taps and hand claps projected on a screen behind the performer (Francie Manhardt—reproduced with permission).

Acoustic rhythms, the regular and patterned marking of the division of time, played on drums, pianos, guitars, in the vocal line and so forth, arise from physical action. Re-embodiment strategies include repetitive pulsing actions with one or more body parts, such as stamping, forward and back motions with a shoulder or the entire upper body, hand beats or head nods. The choices that are made, echo the role of rhythm in the source material. For songs with a persistent, dominant beat, the motions may be largely continuous. A Visuele Muziek student presentation in 2020 used a split screen video projection of the performer tapping her foot to the main beat and clapping hands to the punctuating rhythm, throughout the song (see Figure 3). In other works, some sections have a more dominant rhythm than others; these “highlights” are reflected in the size of particular movements and the extent of body involvement, and by the performer guiding the audience’s attention through their eye-gaze.

Rhythmic sounds are predominantly embodied but occasionally an interpreter will enact the source of the sound, such as through imitating a drum solo.

It is important to note that d/Deaf interpreters are less likely than hearing interpreters to continuously embody the pulsing rhythm of a song (Maler, 2015). This is because non-manual markers, including head and torso movements, play an important role in sign language prosody^24^ (Pfau and Quer, 2010)—co-opting these body-parts for the demonstration of rhythm interferes with their prosodic use.

Rhythm in the vocal line is often informed by the innate rhythmical patternings of lyrics, which arise from the division of words into syllables. Sometimes these are modified to create poetic patterns, such as extending the sounding of words over numerous counts. For example, the words “down” and “out” in consecutive lines in “This is Me” (Pasek and Paul, 2017) are held over three counts.^25^ Manual signs, also have their own innate rhythms which may be modified to echo musical phrasing. For example, when lyrics are extended temporally as with “out” in “This is Me,” the sign/gesture “stroke” (the meaningful section of a sign unit) may be extended in both time and space. This is shown in Figure 1 in which Stolk prolongs the gesture over three counts and moves her hands beyond the standard gesture space, into the extreme periphery.

When a single sign encapsulates a multi-word phrase, one solution is to parrot the rhythm of the lyrics by repeating the signed action. For example, in Sarah Tubert’s official ASL version of Show Yourself Show Yourself (From “Frozen 2”/American Sign Language Version), 2020 the lyrics “come to me” are signed using the single, repeated movement of one hand after the other, drawing toward the interpreter’s heart, like beckoning someone in. Rhythmically, this is similar to the sung lyrics but uses one repeated, complex gesture instead of three separate words.

An alternative solution is to let the sign language rendition have its own patterning, matching the source song only in terms of phrase length and overall dynamic qualities (for examples, see Amber Galloway Gallego’s YouTube channel—see Table 1). All three of these strategies may be accompanied by a pulsing motion with part or the whole body to maintain the underlying musical pulse, which emphasizes the framing of the action as being within a song (Maler, 2013).

**Discussion:** rhythmic sounds are often mapped to rhythmic bodily movements with the size and effort of motion frequently reflecting the dominance and power of the sound within the mix. Time is also often mapped to space, with more time taking up more space.

As was noted in the background theory, rhythm and other aspects of the division of time are amodal concepts that can be marked in different modalities. Regular, on-going rhythmic pulses are fundamental to life: heart-beats, breathing, walking, tapping, shaking and swinging are just a few examples that permeate our lives.^26^ Such rhythmic stimuli may be experienced internally through proprioception^27^ or from external signals, involving sound, vision or touch. We know what they feel like when we experience them in our own bodies, and can empathize with them, when we observe them in others.

Additionally, sound itself is essentially a physical phenomenon arising from the vibration of particles within solid, liquid and gaseous substances which create compression-decompression (acoustic) waves. These can be perceived by hearing humans when they reach the ear-drum with frequencies within a particular range, before being transmitted to and decoded by the brain. The physical vibrations that are decoded as sound can also be experienced through other parts of the body via the somatosensory system although the ability to interpret complex features of sound through physically felt input is limited.^28^ Tactile sound has been found to be processed in the same parts of the brain as auditory sound (Levänen and Hamdorf, 2001). d/Deaf people can be particularly attuned to felt rhythm especially through resonant materials such as a wooden floor or a speaker placed inside clothing (da Fonseca-Wollheim, 2021), which visual marking can enhance.

Sound sources produce complex acoustic waves known as amplitude envelopes that form quasi-rhythmic clusters at different timescales such as syllabic and phrasal in speech, or note and phrasal in music (Pouw et al., 2021b, p. 12).^29^ Co-speech and musical gesture research studies have shown that such energy pulses can be echoed or co-ordinated across modalities, for example with accompanying movements. Whilst there is often measurable interaction between, for example, words and gestures, timing may not be in unison—the acoustic and embodied forms may follow independent, intrinsic patterns, informed by the physical constraints of the producing entity (e.g., instrument or body) and/or conventions such as grammatical structure of spoken or sign language. This may have an impact on the temporal features of an embodied song, with interpreters making choices about which system leads the timing—both how the embodied song relates to the acoustic version but also, how music-like embodiments interact with the sign-linguistic features [see also, sections on “mouthings” and “individual differences” (section “Interpreters’ Identities as Broad Context”)].

Neurologically, “perception of both auditory and visual rhythms shares neural substrates with the motor system in terms of timing mechanisms” (Pouw et al., 2021b, p. 8—based on research by Schubotz et al., 2000). Synchronization of rhythm between two entities, particularly with respect to body functions and processes, such as tapping along to music or brainwave frequencies aligning with a periodic stimulus (regular rhythm), is known as entrainment (Korsakova-Kreyn, 2018). Rhythm in sound and observed movement can illicit entrainment in the audience, which can feel good physiologically and socially (feeling in-tune with the crowd at a concert) and can add to the meaning of a song by evoking imagery associated with the pacing or patterning of the rhythmic marking (Carlson et al., 2019; Marshall-Rose, 2019; Singer, 2019).

Beyond steady pulses, we have embodied and multisensory knowledge of other divisions of time that can be evoked across modalities, which can all inform our understanding of durational information that is communicated in song form, regardless of the modality. These include sensory-motor knowledge of patterned and irregular temporal phrasing, and acceleration and deceleration, that is, the shortening and extending of gaps between markers of time, such as the feel, sound and vision of a car leaving and approaching traffic lights.^30^ Some markers of time experienced in the world produce sounds, many have spatial dimensions and involve some form of action; they all share an underlying amodal structure.

#### Intensity and Other Qualitative Features

Intensity—the amount of something per unit—can apply to musical features such as loudness,^31^ complexity and tension, and specific concepts such as crescendo. High intensity is often embodied as shaking motions within articulators, fast and/or large motion and the involvement of more of the body, including the face. Thus, a crescendo is often shown through tense vibrations of many articulators, whereas pianissimo (soft, gentle playing) is often suggested by small, gentle, relaxed movements with attention on a single articulator.

**FIGURE 4:**
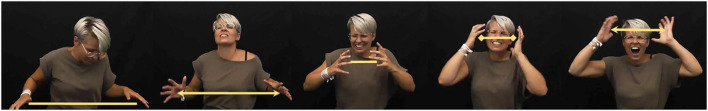
Embodiment of instrumental crescendo—increase in energy in the music, transformed (left to right) into tension in the upper-body, drawing the hands inward and upward until it explodes in a scream-like release (Stolk: Pyscho—Muse, 2020).

An example of an embodied crescendo occurs in the instrumental break in Stolk’s version of “Psycho” by Muse (2020; Figure 4). Throughout the 30 second increase in intensity, Stolk’s hands are tense and claw like, fingers shaking, Beginning palm down at waist height, out to the sides of her body, they rotate first to the audience and then draw toward each other, hands journeying up and in, toward her face; her body is swaying side-to-side with the beat of the music but becomes more frantic and more extreme; elbows pulse in and out as if pumping at bellows, her face tenses up, eyes mainly closed, until her mouth opens in a silent scream.

This example illustrates the integrated nature of emotional expression within embodied song interpretations, involving the whole body, including facial expression.

**Discussion:** intensity of sound is embodied through muscle tension, the height and size of motion, and involvement of more/less articulators, including the face. Intensity has been recognized as an amodal structural concept (Stevens, 1957; Spence, 2011) and each modal system uses the tools available to communicate the level and type of intensity relative to other features. Thus, combinations of more actions, use of more space, more power, tension, velocity and acceleration/deceleration (such as in shaking movements), and the involvement of more body parts, imply increases in volume, complexity (e.g., rhythm and instrumentation), and emotion.

The observation that volume tends to be reflected in amplitude (size) and speed of movements has also been noted in experimental data (Godøy, 2010). Further investigations could reveal if some mappings are more common (production and corpus research) or create a better fit than others (comprehension studies).

In general, the performative and expressive context of a song often places greater emphasis on dynamic and qualitative features such as intensity, tension and power, than is present in everyday spoken/signed interactions. Meaning is established in part through nuances of these effort qualities in both acoustic and embodied-visual song forms. Sound and movement dynamics are both difficult to communicate in words but the direct transformation of the “feel” or energetic features of music into bodily movement do not require language. It is perhaps easier for some people to embody sound quality than to describe it linguistically. Similarly, an observer can have a kinaesthetic response to embodied sound quality, that is, they can “understand” it viscerally. Such bodily understanding may be independent of knowledge or prior experience of the acoustic source.

#### Timbre and Instrumentation

Timbre is the “quality” of sound specifically arising from the frequency spectrum (range of harmonics) and envelope (changes in amplitude and other features over time) produced by a particular instrument, including the voice. It is also known as tone color or quality.

**FIGURE 5:**
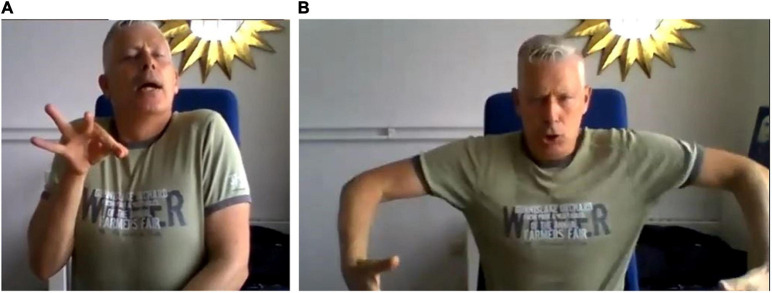
Embodiment of instrumental quality (timbre)—**(A)** long, thin and delicate violin vs. **(B)** “not violin” (André Uittenbogert—reproduced with permission).

Timbre plays an important role in establishing the “feel” of a song. It is embodied in complex ways. André Uittenbogert gave the example of the sound of a violin being “long and delicate”—a description accompanied by demonstration—one hand moving up the center line of the body, pulling away from the other hand, with a pinched thumb and forefinger, like pulling a thread. In addition, his whole body squeezed toward the center line and rose slightly upward (see Figure 5 shown in contrast with “not violin” movement). As in this example, embodiment of timbre draws on effort qualities, sometimes integrated with a visual indication of pitch.

Instrumentation can also be shown through using a standard sign for the instrument, which often coincides with (or is similar to) the action of playing that instrument. That is, using enactment. Such enactment does not, in itself, communicate anything about the timbre of the sound, which is a feature that many d/Deaf audience members cannot directly perceive. The Visuele Muziek training discourages this strategy, guiding student performers to embody the quality of the sound, translating the “feeling” into movement rather than mimicking the action of playing the instrument.

There are interpreters who choose to combine an indication of the way an instrument is played with the rhythm and “feel” of the sound. This is hinted at in Stolk’s opening of “This is Me” discussed below, and is even more explicit in Galloway Gallego’s rendition (2018) in which she begins as though playing an imaginary piano in front of her. She then raises her right hand as the tone rises and marks out the single notes as they are played. Meanwhile, her facial expression suggests paying close attention to the sound with emotional intensity (a conventional element of sign language prosody). Her left hand is held in place, echoing the accompanying held chord sound. Such “air-instrument” style imitation combines enactment with embodiment of the musical quality, “the dynamics, i.e., the speed, acceleration, force, effort” (Godøy, 2010, p. 113), and provides a visible indication of the emotional resonance of the instrumental line.

Sound effects are a rarely used but interesting feature, linked to timbre and instrumentation. Their acoustic signal arises from, or references, an event that has a physical origin, such as breaking glass. Acting out the action of a glass dropping and shattering (embodiment of the act of shattering), simultaneously references the aural and visual features of the source event. For hearing people, the sound and vision are connected in formative experiences. Deaf people may have mono-sensual knowledge of breaking glass and the interpreter’s role is to fill in a gap by making visual reference to relevant knowledge.

**Discussion:** timbre is embodied through effort quality, use of space, facial expression and non-manual gestures, sometimes combined with enactment of “playing an instrument.”

The timbre of a sound is a product of physical characteristics of an instrument and forces acting on it. Dominant features are the size and shape of any resonating chamber; length and tension of taut materials such as strings and skins; and the materials from which they are made. Because of their physical nature it is possible to experience the vibrations of “sound” through the somatosensory system and this felt sensation of sound could inform embodied representations, with interpreters extending or exaggerating the felt qualities to make them more accessible for d/Deaf viewers. This is an example of correspondences arising from a common source—the physical attributes of the instruments give rise to vibration patterns that can be both heard and felt.

#### Pitch and Melody

**FIGURE 6:**
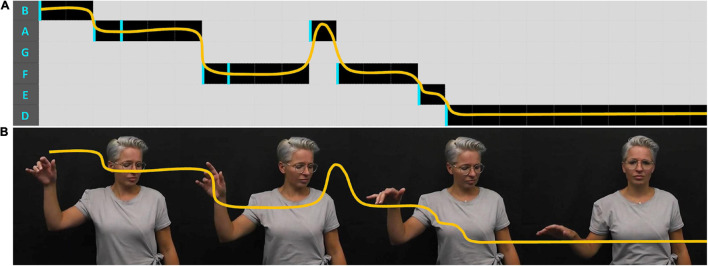
Spatial representation of the melodic line—**(A)** shows a spatial visualization of the melodic line—height placement represents the note (as on a musical score), width represents time, progressing from left to right, onset of a new piano key-press indicated by a vertical line. **(B)** Shows the melodic line represented by movements of the right hand, over time (Stolk: This is Me—Greatest Showman, 2020).

Pitch and melody (changes in pitch over time) are most often made visible in instrumental sections, such as intros and tend to be represented spatially. For example, Stolk embodies the opening piano melody in “This is Me” by raising her right hand in space and lets it float back down, like a falling leaf, the descent punctuated by little twists and shifts in handshape, hinting at the press of piano keys, rhythmically shifting down a scale (Figure 6).

Pitch relations are predominantly integrated into a musical discourse, that is, they are sung or played on instruments with particular qualities using rhythmic and prosodic-like intensity patterning. Similarly, embodied representation of pitch and melody is integrated with other features. Rather than schematized renderings, melody representations may draw on, or appear reminiscent of, idiosyncratic imagery. Evocation of familiar experiences may help ensure relevance of a potentially meaningless concept for d/Deaf audiences.

**Discussion:** Pitch is an attribute of sound—a term that refers to the frequency of an acoustic wave—existing only in the aural modality. There is, however, a great deal of research into CMC of pitch, for example, Eitan and Timmers (2010) identified and investigated 35 pitch-mappings across diverse cultures. Many CMC have spatial properties such as height and thickness. Research most commonly investigates correspondences for *relative pitch*, such as “high/low” contrasts, scales or melodies.

The association between pitch and spatial metaphors has been investigated in psycholinguistic studies such as by Dolscheid et al. (2013). They demonstrated that specific associations which are separately dominant in different cultures—high/low in English and Dutch vs. thin/thick in Farsi—occur at a cognitive, non-linguistic level, and, for Dutch speakers at least, the alternative conventional framing is also favored (“thin” being more accepted than “thick” as a visual descriptor for high frequency pitches). It has not yet been definitively established how these associations form, whether they are somehow innate (structural) or socially learnt (statistical).

In an endeavor to test whether the CMC of pitch relations are conceptual (linked to a deep, shared structure) rather than perceptual (direct association between sensory-motor experiences), Antović et al. (2020) carried out a series of experiments. They found that participants rated diverse visual representations (vertical movement, size change, width and rotation) as similarly appropriate analogs to scalar pitch change, without favoring the culturally dominant semantic form (vertical movement). This suggests that the ubiquity of “high/low” in some cultural discourses, is semantically mediated but that underlying structural correspondences take precedence in non-linguistic settings. They proposed that underlying abstract schematic features inform congruency, in particular “discrete distance” (step-wise changes) and “scalar change” (directional progress). They noted additionally, that multi-feature analogs were reported to be a better fit than those that mapped only a single feature. From a structure mapping perspective, any entity or representation in which the elements relate to each other the same way—stepwise, directional changes—can be understood as being analogous. This supports the observation that for melody embodiment in song interpretations, an analogy is established between “the relationship between the acoustic notes” and “the relationship between positions in vertical space.”

Whilst this research provides evidence that CMC for pitch relations are at least in part established structurally, it may also be the case that certain representations are preferred due to statistical correspondences. That is, they are experienced in nature/the world, more than others, which may in part be because of a causal relationship. For example, most birds produce high frequency tones due to the size and shape of their vocal apparatus (causal co-referent correspondence) they also tend to exist high in vertical space (common co-referent correspondence).

Initial observation suggests that visual representations of pitch and melody are not pervasive in embodied songs. A more thorough survey could establish the extent to which they are considered salient and if this differs between hearing and d/Deaf interpreters. Additionally, differences in strategies could be explored in relation to cultural diversity and the role of intermediary imagery (analogies), in both production and comprehension.

#### Musical Features Conclusion

The musical features of a song work together to enhance the meaning constructed by the lyrics. Diverse and complex strategies are often involved in embodied interpretations, combining visual amplification of somatosensory information, and visual representation of both amodal features such as intensity, and mode specific elements like melody. Imagery is often evoked by the specific combination of temporal, spatial and dynamic features, and this may feed into and enrich potential meanings.

To illustrate this combination of structural equivalence and the augmentation of meaning through analogous imagery, arising from a complex acoustic unit, I will return to Stolk’s introduction to “This is Me.” I will use the analogy structure mapping framework to compare the melody and its associated rhythm, with the embodied rendition, and the “falling leaf” image that it evoked (author’s interpretation, see Figure 6). This is presented as an indicative analysis, to illustrate how a close reading using structure mapping may help to unpeel how cross-modal meaning-making arises. More robust follow-up studies are advocated, using quantitative kinematic analysis tools such as those mentioned in the general discussion.

The comparable ATTRIBUTES in each analog are hand and leaf positions in vertical space, and musical notes on a diatonic scale. The former two share visually iconic similarities of shape/space. The “falling leaf” is a mental simulation that *can* behave in the ways indicated.

Comparison of STRUCTURAL RELATIONS involves attention to the patterns that the attributes follow over the timescale of the “event,” that is, how one note or hand/leaf position relates to another over time. Categories of “directional” change, rhythm and dynamic qualities are pertinent in all three analogs. The “vertical direction” and extent to which the attributes “move” over time is roughly equivalent. Whilst spatial, metaphorical language is commonly used to describe musical features, the changes being noted are in the wavelengths/frequencies of the acoustic tones, which change in stepwise patterns that are analogous to the spatial pathways. Changes of position or tone within all three variants follow a similar rhythmic pattern and each ends with a hold. Dynamically, rhythmic marking arises from small bursts of energy (patterns of acceleration/deceleration), which are paralleled across the domains. All three referents can be generally characterized as light and calm throughout.

At an AFFECTIVE level, all these analogs inspire a sense of calm and anticipation, the latter aspect potentially arising from the held note/spatial position which invites resolution. The integrated features of each provide a structure that can carry across to more METAPHORIC INTERPRETATIONS of “something delicate settling into place” or the more abstract “finding self-worth.”

The **local context** of the segment at the start of the song, introduces key themes and sets the tone. This placement will influence interpretations.

**Role and contribution of imagery:** knowledge of the physical properties of a falling leaf, how it twists and turns in air currents before coming to rest, as well as its association with the seasonal maturity of Autumn, inform my interpretation of the observed movement. Other, similar images, such as “water flowing along a stream, reaching a stretch of calm,” can also feed into interpretation. Different images will be triggered for different people and will influence their response to the song. Conscious use of imagery in production will offer specific details that may guide viewers’ interpretations. The embodiments chosen will be informed by how the interpreter perceives the acoustic stimulus, including the images that it evokes for them from their own prior experiences.

**FIGURE 7:**
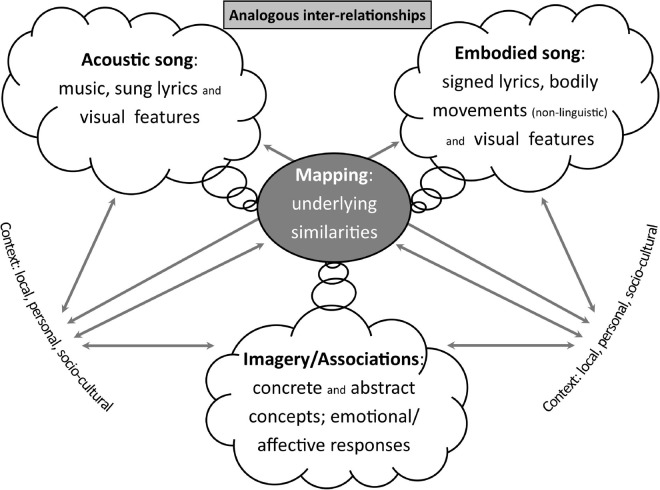
Analogous inter-relationships—analogies in different modalities are linked by the mapping of their underlying similarities; choices and attention are influenced by context.

**Summary:** Aspects of the acoustic song, embodied interpretations, and other imagery and associations (including triggered emotional responses) are mapped/connected via shared structural similarities. Context shapes and informs choices and meaning-making, and is a key factor in understanding diversity of interpretation (see Figure 7). This is expanded upon in section “Interpreters’ Identities as Broad Context.”

## Interpreters’ Identities as Broad Context

A range of potential strategies have been discussed, which offer a broad framework of possibilities for embodied song production. An interpreter uses the capacities of the tools available to them, that is, their body and its movement range, within the confines of the signing space. Choices are also constrained by rules and practices of the sign language with which the music-like movements are integrating. A dance interpretation of a song would be shaped by different constraints, such as the size of the performance space, greater use of three-dimensionality and whole-body movements. The goal may be less tied to a desire to communicate the “same” meaning, allowing for more diverse responses to the musical stimulus.

The production of embodied song interpretations draws on both fundamental connections between movement and sound, and the sensorimotor grounding of meaning associations within personal and cultural experiences. Beyond largely shared linguistic-cultural influences, interpreters’ individual characters, styles and “readings” of songs, shine through the patterns that emerge in their practices. For example, Sarah Tubert uses clear, strong handshapes with noticeable muscle tension, her movements are predominantly percussive, her body upright and centered and eye-gaze largely directed toward the camera, she primarily combines full grammatical ASL with song-synchronous English mouthings. Mirjam Stolk pulses her body along with dominant rhythms and echoes melodic, instrumental lines with hand gestures; she signs using grammatically rich NGT, but this is mainly structured to coincide with the word order of the sung lyrics, which she (partially) mouths synchronously in the original language of the song. Amber Galloway Gallego, gives highly impassioned performances, predominantly using sign language word order and rhythmic phrasing, with linked mouthings, and integrating embodiment of the instrumentation. These examples give a taste of how the individual behavioral patterns of an interpreter may be more distinctive than musical genre.^32^ This follows Carlson et al. (2020) who demonstrated using motion capture that individual dancers have kinematic signatures that remain consistent across music genres and are much more pronounced than shared genre specific movement responses (that is, a music genre having a consistent movement signature across dancers). In this way, an interpretation is like a “cover version” of a song in a different modality, in which the practitioner makes stylistic and artistic choices.

Personal style can be understood as **broad context**—the habits, skills and values that inform interpretive choices. Within the practice of embodied song interpretation, there is a particularly interesting phenomenon in the relationship between d/Deaf and hearing creative partners who work together to create an embodied interpretation. This Performer/Feeder relationship provides a complex, amalgamated knowledge and skill base (summarized in Supplementary Appendix 2). The interactions within these creative partnerships offer a rich and unique resource for cross- and multimodal communication research.

## General Discussion

Embodied song interpretation is a highly complex and unusual paradigm through which we can gain insight into the meaning of “meaning” and how it can be communicated across modalities.

This paper started by noting that a song (in acoustic or embodied form), is a gestalt in which meaning emerges from the integration of a wide range of features enhancing the message imparted by the lyrics. In order to unpack the types of information that can be communicated in each modality and how they relate to each other, I separated the forms into constituent parts, according to the framing proposed by practitioners in the Netherlands.

Whilst the examples and discussions focussed in on the individual features, such as rhythm, the meaning associated with each instance emerges from the specific interactions within and between features. For example, the impact of a particular rhythm within a particular song is established by qualitative details and the local context. These include features such as the instruments or articulators used, its dominance in the (acoustic or visual) “mix,” and how much energy is expended. Changes to any of these elements changes the “feel” of the rhythm, invokes different imagery, and ultimately alters the meaning.

Embodied song practices assume that embodied interpretations of songs provide d/Deaf audiences with information that is “meaningful” in some equivalent way. For the connection between the signer’s gestures and music to go beyond a sense of conformance (as proposed by Maler, 2013, p. 3.7) they must communicate equivalent conceptual information and be likely to trigger the same emotional responses. In this paper, I have begun to unpack the relationship between the signals in the two modalities to suggest how this may be established.

The emerging hypothesis is that meaning arises in embodied songs through shared patterns of internal relations with musical forms and sensory-motor referents in the world, across a range of amodal and cross-modal features. Dynamic features appear to play a dominant role in establishing and refining meaning, especially informing affective responses. Although linked to dynamic qualitative features, further research is particularly needed into how the “feel” of sound and movement correspond.

As humans we have an innate potential to recognize underlying patterns and deep structures regardless of the context or modality (i.e., structural correspondences) but we primarily engage with, understand and communicate these through reference to sensory-motor experiences which can be highly specific (i.e., statistical correspondences). Patterns between elements within a system, such as rhyming words, interacting instruments or melodic patterns within an acoustic song find analogs such as rhyming signs, interacting articulators or spatial patterns within embodied songs. Within their respective forms, these work together to create meaning through association with prior experiences.

The explorations at the heart of this paper begin to suggest in what ways some analogies fit better than others but they also reveal that far more work needs to be done to expand our understanding of how embodied and grounded experiences enable us to understand the world.

### Future Directions

I will conclude this paper with some thoughts about potential studies into embodied songs, to expand our understanding of para-linguistic meaning-making within a broadly grounded cognition-based framing. I divide these into two broad and overlapping approaches—investigating “meaning” comprehension, and analysis of form—that draw on diverse fields of study, and include both quantitative and qualitative methodologies. The involvement of d/Deaf researchers in study design, implementation and analysis would ensure that sensory atypical experiences and insights inform and enrich our developing understanding of embodied meaning-making.

In general, a diverse range of subject populations could valuably be engaged, depending on the specific research questions. These include hearing, d/Deaf and hard of hearing participants with different levels of sign language expertise, as well as embodiment specialists such as dancers from across the deaf-hearing spectrum.

#### “Meaning” Comprehension

Embodied song interpretation practices inspire a range of research questions associated with perception, processing, comprehension, and sensory-diversity. For example: What are the similarities and differences in both conceptual and affective comprehension of embodied songs in d/Deaf and hearing viewers? Do hearing viewers perceive acoustic and embodied-visual versions as being analogous (in what ways)? In what ways do changes to feature parameters influence perceived meanings? What neural pathways/brain regions are involved in the processing of acoustic and embodied signals in comprehension?

Establishing methodologies that combine ecological and scientific validity remains challenging but Jola et al. (2012) have presented a valuable discussion of the integration of qualitative, phenomenological, and quantitative neurophysiological methodological approaches to the study of dance that can help guide research going forward. Potential methodologies include eliciting responses to both music and embodied-visual stimuli (combining traditional production and comprehension experimental paradigms). Data collected could include embodied interpretations recorded using video and motion capture, as well as written, spoken and signed, forced choice and open description responses. Response prompt examples include:

◼ **Open:** “This movement (or piece of music) is like/reminds me of/makes me feel…”
◼ **Restricted choice:** “This [stimulus (movement or music phrase)] is most like: [(choice of) word/sign, movement or music phrase].”

Match/mismatch methodologies can be employed, for instance pairing different music and movement phrases and can include manipulation of specific variables such as dynamic qualities of weight or flow in movement execution. Priming tasks which reveal perceptions and processing of (in)congruency (i.e., agreement) between a prime and a target can be extended beyond traditional linguistic contexts, garnering both behavioral and neurological data such as in EEG (ERP)—N400 responses (Amoruso et al., 2013). There is also scope for investigating similarities and differences in neurological responses, such as potential involvement of the sensorimotor system, when watching, comprehending and thinking about embodied song interpretations. This could include differentiating between kinaesthetic motor and visual imagery and associated mental simulations (Jeannerod, 2001; Humphries et al., 2021, p. 4). Brain imaging study design is, however, constrained by the sensitivity of the technologies to interference from movement.

Uni- and multimodal data can be analyzed using measures of conceptual closeness, semantic mapping, and distributional semantics and kinematics (Bolognesi and Aina, 2019; Pouw et al., 2021a) to provide insight into networks of meaning, within and across modalities. For embodied data this requires some unpacking of the physical (including spatial and dynamic) form, as is discussed in the following section.

#### Mapping of Features Across Modalities—Analysis of Form

The overarching aim of analyzing acoustic and embodied-visual song forms is to unpack the shared patterns of internal relations across modalities. The core challenges to this is in developing signal specific methodologies to measure and compare salient features and relations. Specifically, challenges exist around the under-defined notion of dynamic qualities in both modalities. Indicative research questions include: (In what ways) do movements associated with related semantic and/or musical units, move similarly to each other? (adapted from Pouw et al., 2021a, p. 2); which features map across modalities and in what ways?

Such questions could be tackled using both experimental (production) and corpora-sourced videos/motion-capture recordings which could be analyzed using multimodal, multidimensional annotation tools such as M3D (Rohrer et al., 2020) an extension of ELAN (Brugman and Russe, 2004; Crasborn and Sloetjes, 2008). This coding system draws together prior protocols to enable analysis of the complex interactions that characterize embodied song interpretations.

Previous studies that can inform future research include Godøy’s (2010) work on embodied descriptions of music, which found that dynamic qualities are more likely to be shared, than geometrical/spatial features or body-part use. For example, different performers may embody the same melodic line of a saxophone, with one shimmying their torso and another snaking their arm in-front of their body, but both would have comparable rhythm and muscle tension. Pouw et al. (2021a; private correspondence) is working on automated analysis of kinematic features such as spatial pathways and velocity profiles (the patterns of increases and decreases in speed) and has proposed a methodology for establishing “kinematic closeness” that parallels distributional semantics and illuminates the broad, distributed nature of meaning that emerges from non-symbolic signs.^33^

#### Additional Questions/Studies Focused on Sensory Diversity

Study of embodied song interpretation practices can also provide insight into sensory awareness and embodiment behaviors across sensory-diverse populations, through answering questions such as: Is foregrounding of tactile features of music common amongst d/Deaf performers? How is meaning made and communicated within the particular sensory constraints of the Performer/Feeder production process?

## Conclusion

In this paper, I have considered the broad notion of meaning-making within the context of embodied song interpretations, drawing on a wide range of theories within a grounded cognition framework. I have proposed that underlying structural forms and relationships enable the creation of equivalent representations across modalities. The specific, detailed and complex embodied forms, and the meanings arising from them are informed by prior sensory-motor knowledge and experiences. Ecologically valid research studies based on embodied song practices promise rich, new insights into the embodied and grounded nature of meaning-making. Embodied song interpretation also has a great deal to offer as a participatory practice, increasing access for all, to the physical musicality of movement and embodied meaning-making.

## Data Availability Statement

Publicly available resources were analyzed in this study. All data are available on YouTube/Facebook as cited in the article. They are individual resources and do not constitute a distinct dataset.

## Ethics Statement

Written informed consent was obtained from the individual(s) for the publication of any potentially identifiable images or data included in this article.

## Author Contributions

The author confirms being the sole contributor of this work and has approved it for publication.

## Conflict of Interest

The author declares that the research was conducted in the absence of any commercial or financial relationships that could be construed as a potential conflict of interest.

## Publisher’s Note

All claims expressed in this article are solely those of the authors and do not necessarily represent those of their affiliated organizations, or those of the publisher, the editors and the reviewers. Any product that may be evaluated in this article, or claim that may be made by its manufacturer, is not guaranteed or endorsed by the publisher.

## Footnotes

1 Although many dictionary definitions of dance refer to movement in relation to music, anyone with experience of Modern and especially Post-Modern dance practices know that music is not an essential factor. For the practitioner, dance can be predominantly about the experience of moving—exploring what is possible, and what it feels like (see for example Overlie, 2006; Tufnell and Crickmay, 2006; Koteen and Stark Smith, 2008).

2 In line with scholarship and customs related to Deaf culture, people with some degree of “medical deafness,” who identify as belonging to Deaf culture will be referred to as Deaf with a capital “D.” Those with medical deafness, who do not identify with the Deaf culture, are referred to as deaf with a lower-case “d.” Some people also refer to themselves a “hard-of-hearing.” When referring to these groups of individuals together, I will use the term d/Deaf.

3 The term acoustic can be used to refer to both sound and the sense of hearing. The alternative meaning of natural as opposed to electronically modified music is not intended.

4 The most common label in United States is Song Signing—see Maler (2015) for a discussion of different forms. In the Netherlands terms used include Muziektolk (Music interpretation), Sign-dancing and Visuele Muziek (Visual Music)—see Supplementary Appendix 1 for further details.

5 As with spoken languages, there are many distinct sign languages around the world, each with their own vocabulary and grammatical rules.

6 It is recognized that not all d/Deaf people are interested in signed song interpretations. It can be argued that a form which gives precedence to a hearing-oriented stimulus is irrelevant and even detrimental to Deaf culture—an in-depth discussion of this important issue is beyond the scope of this paper.

7 A signal can be defined as “A measurable aspect of a producing system, changing in time” (Pouw et al., 2021b, p. 6, Box 1).

8 Cross-modal is sometimes written without a hyphen, echoing the common spelling of multimodal. I have elected to use the hyphenated version to emphasize the compound nature of the term.

9 An alternative or complementary approach is to focus on the embodied-visual form as an autonomous entity, irrespective of any acoustic precedent.

10 The term sensory-motor is used to denote inputs and processing involving any part of our sensory and motor systems as opposed to the integrating sensorimotor system.

11 “Kinaesthetic experience” refers to the felt sense of movement.

12 Extensive debate exists around whether, or in what ways, music is referential and/or more broadly meaningful. This paper takes the position that within the context of songs, all musical features work together to create a coherent whole, and are thus intrinsically meaningful—they in some way help the song “mean” something.

13 Various formulations of the modes have been proposed, this list is taken specifically from Cienki 2017.

14 Adjustments such as these have been identified in relation to co-speech gestures performed for communicative effect, for example in adverse sound conditions (Trujillo et al., 2018).

15 The two-tiered use of the term embodiment is recognized as being potentially problematic. All bodily movement involved in embodied song interpretations are considered to be embodiment, however, within this broad category, lies the more specific sub-category of embodiment in terms of moving “as if” you are another entity.

16 These terms do refer to slightly different phenomena but in the context of this paper, they will generally be used interchangeably.

17 As is the convention in Conceptual Metaphor Theory, I have used SMALL CAPITALS to denote (SOURCE and TARGET) concepts.

18 Synaesthesia is a specific, rare form of structural CMC in which stimuli or concepts generally associated with one sense are (usually additionally) experienced via another, such as seeing music in color (Ramachandran and Hubbard, 2001; Cuskley et al., 2019).

19 The neural basis for structural correspondences may be associated with the extensive multimodal connectivity that is a (potentially) unique characteristic of human brains (Ardesch et al., 2019).

20 Feeling like you are experiencing the movements and emotions of others, is known a kinaesthetic empathy and likely involves regions of the brain known as the Action Observation Network (AON), which includes the posited Mirror Neuron system (Jeannerod, 2001; Hagendoorn, 2004; Calvo-Merino et al., 2006).

21 This may be done as part of a formalized system such as Sign Supported Speech, Sign Assisted Language and Signed Exact English, or may occur more idiosyncratically.

22 Nederlandse Gebarentaal—Dutch Sign Language.

23 Super Linguistics primarily employs formal linguistic methodologies but also offers rich possibilities for cognitive linguistics and embodied cognition.

24 Prosody refers to features of a language such as intonation, stress and rhythm, which reflect and communicate information such as the emotional states and intention. It pertains to the way that things are “said” or “signed.”

25 In addition, the whole of each preceding phrase: “When the sharpest words gonna cut me down. I’m gonna send a flood, gonna drown them out,” follows almost identical melody and rhythmic patterning (Pasek and Paul, 2017, p. 3).

26 The musical training system: Dalcroze Eurythmics, “introduces a musical concept through movement,” focussing on rhythms such as walking as the root of musicality (wiki).

27 “Proprioception” is the internal sense of the position and relationship of parts of the body.

28 A range of terms are employed across the diverse literature related to this field, the broadest being somatosensation, pertaining to the system that receives and interprets sensory information from joints, muscles, and skin. Other relevant terms include “tactile” and “haptic” which both refer to the perception of touch.

29 See also “timbre” for a discussion of amplitude envelopes.

30 All mention of multi-sensory input recognizes the importance of sensory diversity—not everyone experiences all the sensations that are noted.

31 Loudness is often referred to as Dynamics in music theory—this is not used here, to avoid confusion with dancers’ use of dynamics to refer to broader qualities of movement (which does not include volume).

32 From the perspective of psychology research, this corresponds with the notion of “individual differences.”

33 Pouw identified velocity profiles as a feature that differentiated “insect-like” from “water-like” performance of shared movement vocabulary in an unpublished study by Fisher (private correspondence).
