# Supplementary material for: Embodied Songs: Insights Into the Nature of Cross-Modal Meaning-Making Within Sign Language Informed, Embodied Interpretations of Vocal Music
Source: Front Psychol. 2021 Oct 22;12:624689. doi: 10.3389/fpsyg.2021.624689 (PMC8569319; doi:10.3389/fpsyg.2021.624689)
Supplement: Supplementary file 1 [file Data_Sheet_1.docx]

**APPENDIX I – forms of embodied song practices in the Netherlands**

**Muziektolk** (Music Interpretation)

**Primary goal:** communication – increasing access for D/deaf individuals

**Practitioners:** qualified sign language interpreters (Bachelor’s degree); currently all such graduates are hearing.

**Primary features:** clear communication of the lyrics using either primarily grammatical sign language, adjusted to communicate implicit meaning in concord with the musical framework, to fit with phrasing and dominant musical features; or using sign-supported/assisted language. The needs of the stakeholders (those that have requested the service) guide the choice of method.

**Supporting features:** Embodiment of dominant musical features such as rhythm and melody to capture/translate the ‘essence’ of a song, for perception through the visual modality.

**Training:** new advanced training programme (from Autumn 2020) focussing on professional practice. Pre-requisites: 2+ years muziektolk or sign-dance experience and certificate in Visuele Muziek.

**Sign-dance**

**Primary goal:** a variable combination of communication and performance (art) for increased access for D/deaf audience members but also expanding the aesthetic experience for all, including the performers.

**Practitioners:** d/Deaf or hearing users of sign language.

**Primary features:** combination of sign language or sign assisted language translation of lyrics, key ideas &/or underlying meaning/message with embodiment of musical features. Personal style or interests of the performer may be relatively dominant.

**Training:** there is no formal sign-dance training programme in the Netherlands; many practitioners have participated in practical workshops and/or Visuele Muziek training.

**Visuele Muziek** (visual music)

**Primary goal:** performance (art) that translates the entire musical experience into a visual form to increase access to, and enjoyment of vocal music for d/Deaf and diverse audiences.

**Practitioners:** either qualified ‘Muziektolkers’ or Sign-dancers.

**Primary features:** cohesive combination of embodied lyrics in clear but poetic form, embodied musical features and complementary visual elements such as lighting, costume and video projections.

**Training:** an established training programme includes exploration of sign poetry, ‘drawing the song’ and dance/movement. Assessed through performance.
