# Supplementary material for: Embodied Songs: Insights Into the Nature of Cross-Modal Meaning-Making Within Sign Language Informed, Embodied Interpretations of Vocal Music
Source: Front Psychol. 2021 Oct 22;12:624689. doi: 10.3389/fpsyg.2021.624689 (PMC8569319; doi:10.3389/fpsyg.2021.624689)
Supplement: Supplementary file 2 [file Data_Sheet_2.docx]

**APPENDIX II – The Performer/Feeder Relationship**


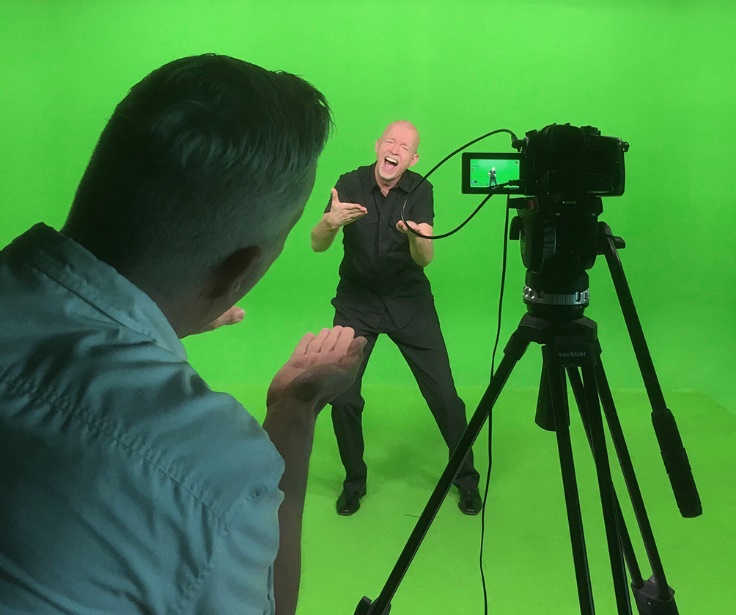


‘Feeder’ - André Uittenbogert & Performer - Tom Uittenbogert

Embodied song interpreters may be anywhere on the d/Deaf-hearing spectrum. Every individual has a different relationship to music and a unique range of skills, abilities and needs. There are features of a song that are not directly perceived by people with degrees of deafness. Their understanding of the stimulus may be mediated through another person’s interpretation of what the sound looks and feels like.^[[1]](#footnote-1)^ In such instances, a d/Deaf performer devises their embodied version from this indirect representation. A hearing person who works closely with a d/Deaf performer to create an interpretation is known as a ‘Feeder’ (personal correspondence with Uittenbogert & Uittenbogert, 2020).

A Feeder provides cues to a d/Deaf embodied song interpreter, to enable them to produce a rich and informative interpretation that is in time with the song. Ideally the pairing involves an almost symbiotic relationship, in which the artists work together to create the piece for performance. Each partner brings both distinct and shared expertise to the process.

In general, a d/Deaf embodied sign practitioner is likely to have relatively deep knowledge and understanding of sign language, allowing for a sophisticated poetic and expressive use of language. Perhaps more importantly, d/Deaf people often have a relationship with visual information and experience of expressing ideas visually via their bodies, that differs to, and likely goes beyond, that of hearing signers.

A Feeder can perceive more layers of information in the source material and has a direct line of communication between these aspects of the music and their bodily response. Features of the music that a Feeder might communicate include instrumentation, pitch, swell and rhythmic cues.

The partners in each ‘team’ negotiate and agree the system that works best for them, within the time-frame available. This may range from basic timing cues, mouthing of the lyrics, conductor-like indication of the dynamic patterns, to a full, behind the camera/un-lit rendition of the entire, mutually created embodied interpretation (see figure above). All cueing occurs slightly ahead of the timing of the song so that the performer will be on, rather than behind, the music.

1. Electronically generated visualisations could offer an alternative route but these too are analogies that map to the source, in this instance, via a mathematical algorithm with parameters selected by the programmer [↑](#footnote-ref-1)
